# Serum Vitamin C concentrations are inversely related to biological aging: a population-based cross-sectional study

**DOI:** 10.1186/s40001-025-03150-w

**Published:** 2025-09-26

**Authors:** Ying Zhang, Fang Gong, Ai-Hua Zhang, Guan-Wu Wang, Yun Liu, Teng Li

**Affiliations:** 1 Department of Laboratory Medicine, Obstetrics and Gynecology Hospital of Fudan University, No.419 Fangxie Road, Shanghai, 200011 China; 2 Department of Hematology, Weifang People’s Hospital, Shandong Second Medical University, No.151 Guangwen Street, Weifang, 261041 Shandong China; 3 Department of Interventional Radiology, Weifang People’s Hospital, Shandong Second Medical University, No.151 Guangwen Street, Weifang, 261041 Shandong China; 4 Department of Radioactive Particle Therapy, Weifang People’s Hospital, Shandong Second Medical University, No.151 Guangwen Street, Weifang, 261041 Shandong China

**Keywords:** Biological aging, Vitamin C, Phenotypic age, Oxidative stress, NHANES

## Abstract

**Background and objective:**

Vitamin C (Vit C), which has antioxidant properties, may reduce oxidative stress and delay aging. However, the relationship between Vit C and biological aging is poorly established. Therefore, we aimed to investigate the association between serum Vit C (S-vit C) concentrations and phenotypic age acceleration (PhenoAgeAccel), an indicator of biological aging.

**Methods:**

The present study utilized a cross-sectional design using NHANES-retrieved data from 10,118 participants aged 20–79. Weighted linear regression models alongside restricted cubic spline analysis were deployed to determine the association. A two-piecewise linear regression model and a log-likelihood ratio test were utilized to assess the potential threshold effect. Using the subgroup analyses, we explored variations in association across different subgroups while utilizing the sensitivity analyses to validate the strength of the outcomes.

**Results:**

S-vit C concentrations were inversely related to PhenoAgeAccel. Participants in the highest quartile of S-vit C levels showed significantly reduced PhenoAgeAccel compared to those in the lowest quartile. A nonlinear relationship was identified between S-vit C levels and PhenoAgeAccel, characterized by an inflection point at 1.46 mg/dL. Beyond this threshold, further increases in S-vit C concentrations did not result in statistically significant reductions in PhenoAgeAccel. The results of subgroup analyses showcased that the inverse association was more substantial among older adults and individuals with hypertension or diabetes. The sensitivity analyses validated the strength of the outcomes.

**Conclusions:**

S-vit C levels exhibit an inverse association with biological aging, particularly in older individuals and those with chronic conditions, highlighting the potential role of Vit C in healthy aging.

## Introduction

Aging is characterized by a progressive decline in physiological functions, leading to increased vulnerability and higher mortality risk (1). It serves as a Major driver for numerous chronic diseases, with age-related conditions contributing to approximately 51.3% of the global disease burden (2, 3). However, the aging process is highly heterogeneous due to individual variability. Chronological age (CA) merely quantifies the passage of time and does not adequately reflect the complexity of aging. Individuals with the same CA often display varying susceptibility to diseases and age-related mortality (4). To overcome this limitation, the biological age (BA) concept has been proposed and is increasingly employed to provide a more accurate representation of the aging process on an individual level (5). Phenotypic age (PhenoAge), a quantifiable indicator of BA calculated through clinical biomarkers, blood cell parameters, and CA, has shown efficacy in identifying individuals at increased risk for various diseases and mortality (6). PhenoAge reflects the CA at which the mortality risk of an individual corresponds to the average risk within a reference population. Phenotypic age acceleration (PhenoAgeAccel) represents the disparity between PhenoAge and CA, where elevated values signify accelerated biological aging (7). Each one-year increase in PhenoAge, adjusted for CA, is associated with an approximate 9% increase in all-cause mortality. And individuals with higher PhenoAgeAccel tend to exhibit a greater burden of chronic diseases, reduced physical function, and impaired cognitive performance (6).

The process of aging involves a complex and multifactorial mechanism, wherein oxidative stress (OxS) serves as a pivotal contributing factor (8). OxS precipitates damage to intracellular proteins, lipids, and DNA, culminating in cellular dysfunction and apoptosis. The progressive accumulation of such damage is a principal catalyst of the aging phenomenon. Furthermore, OxS triggers the activation of inflammatory pathways, thereby inducing a chronic, low-grade inflammatory condition termed “inflammaging,” which further exacerbates cellular damage and accelerates aging (9, 10).

Research has shown that diets abundant in antioxidants can reduce OxS, thereby decelerating the aging process and diminishing age-related disease risks (11–14). However, these studies primarily focus on the effects of specific foods or dietary patterns on aging, and they do not provide clear evidence regarding the precise impact of individual nutrients and their concentrations in the body on the aging process. Moreover, the data obtained through dietary recall are prone to recall bias, which can affect the accuracy of the results.

Vitamin C (Vit C), a water-soluble vitamin that widely exists in vegetables and fruits, possesses diverse antioxidant and anti-inflammatory properties (15, 16). The consumption of Vit C is advocated to prevent and manage age-related conditions (17). Nonetheless, the relationship between Vit C and biological aging remains inadequately investigated. Accordingly, we examined the connection between serum Vit C (S-vit C) concentrations and PhenoAgeAccel in the U.S. population, utilizing the National Health and Nutrition Examination Survey (NHANES) data.

## Methods

### Study population

The NHANES is a continuously conducted, nationally representative, cross-sectional study Managed by the United States Centers for Disease Control and Prevention to ascertain the Health and nutritional status of the noninstitutionalized civilian population in the U.S. Utilizing complex multistage sampling techniques, nearly 10,000 individuals are chosen biennially to participate in comprehensive evaluations. These evaluations encompass the collection of demographic data, biochemical and nutritional analyses, physical examinations, and lifestyle surveys. The https://www.cdc.gov/nchs/nhanes/ website offers detailed data on the survey's design, participant recruitment, and methodology. The National Center for Health Statistics Ethics Review Board approved the NHANES protocol (Protocol #98–12 for 2003–2004 cycle; Protocol #2005–06 for 2005–2006 cycle; Protocol #2018–01 for 2017–2018 cycle), with informed consent being signed by all participants. This study was exempt from approval by our institution's ethics review board due to using publicly accessible and completely de-identified data. Furthermore, the study followed the Strengthening the Reporting of Observational Studies in Epidemiology guidelines (18).

**Fig. 1:**
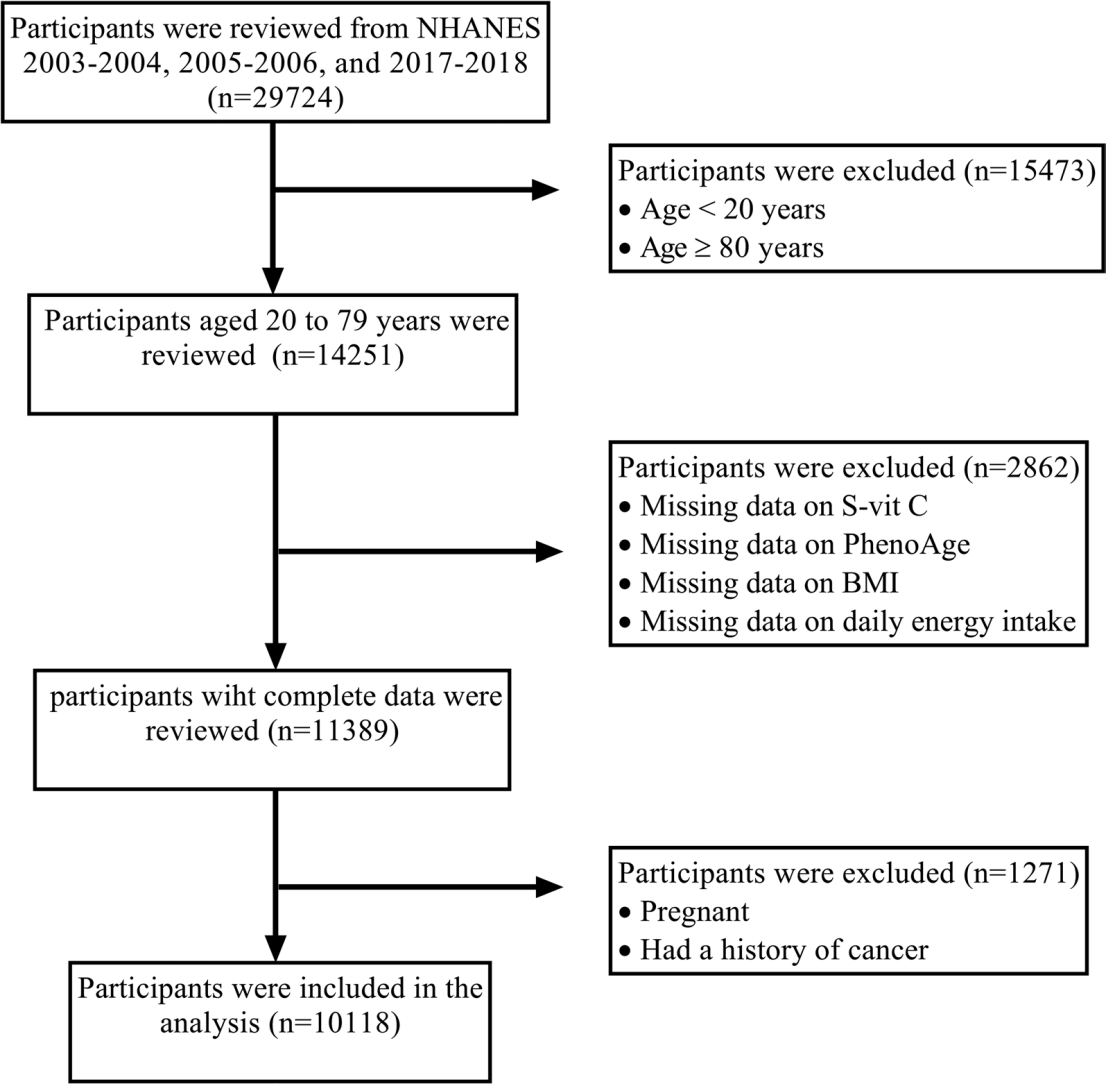
Flowchart of the participant selection process

Herein, we employed three cycles of NHANES data (2003–2004, 2005–2006, and 2017–2018), as they were the only ones providing information on S-vit C concentrations and the necessary data to calculate PhenoAge. In the 2017–2018 cycle, participants at the age of 80 years and older were top-coded at 80 years; consequently, all the analyses were confined to participants aged 20 to 79 years. Exclusion criteria included missing data on S-vit C levels (*n* = 1551), PhenoAge (*n* = 579), body mass index (BMI) (*n* = 112), or daily energy intake (*n* = 620); pregnancy (*n* = 439); and a history of cancer (*n* = 832). In total, the study included 10,118 participants (Fig. 1).

### S-vit C concentrations

S-vit C concentrations (mg/dL) were determined through isocratic ultra-high-performance liquid chromatography with electrochemical detection. Quantitative analyses were performed by comparing the peak areas of serum samples to those of a calibration solution with a known Vit C concentration. Comprehensive guidelines for specimen gathering and processing can be accessed through the NHANES website.

### PhenoAge and PhenoAgeAccel

PhenoAge was derived from CA and nine key biomarkers that reflect critical aging-related processes, including metabolism, immune function, liver and kidney health, nutritional status, and erythrocyte dynamics. These biomarkers include: albumin, glucose, creatinine, C-reactive protein, alkaline phosphatase, lymphocyte percent, red blood cell distribution width, mean cell volume, and white blood cell count, integrated using the following equation (6,19):

$$\text{PhenoAge}=141.50+\frac{\text{ln}[-0.00553\times \text{ln}\left(1-\text{M}\right)]}{0.09165}$$

where

$$\text{M}=1-\text{exp}(\frac{-1.51714\times \text{exp}\left(\text{xb}\right)}{0.0076927})$$

and

xb =  − 19.907 − 0.0336 × albumin + 0.1953 × glucose + 0.0095 × creatinine + 0.00188 × alkaline phosphatase + 0.0954 × ln (C-reactive protein) − 0.0120 × lymphocyte percent + 0.3306 × red blood cell distribution width + 0.0268 × mean cell volume + 0.0554 × white blood cell count + 0.0804 × CA.

PhenoAgeAccel represents the residual obtained from a linear model regressing PhenoAge on CA. This metric reflects PhenoAge after accounting for CA and indicates whether an individual is physiologically younger (negative value) or older (positive value) than expected.

### Covariates

The analyses incorporated a range of potential covariates: age, sex, race, marital status, education level, household income, diabetes, hypertension, smoking status, alcohol consumption, BMI, energy intake, Vit C supplements use, dietary inflammatory index (DII), and physical activity. Based on the NHANES survey, race was classified into non-Hispanic White/Black, Mexican American, other Hispanic, and other races. Meanwhile, education level was stratified into < high school, high school graduate, and > high school. The classification of marital status was as married/living with a partner, never married, or widowed/divorced/separated. Household income was evaluated utilizing the poverty income ratio and classified into low (< 1.30), moderate (1.30–3.50), and high (≥ 3.50) categories. Diabetes was defined by fasting blood glucose ≥ 126 mg/dL, HbA1c ≥ 6.5%, a self-reported diabetes history, or the use of oral hypoglycemic medications/insulin. Moreover, hypertension was identified in accordance with the 2017 American Heart Association/American College of Cardiology guidelines, which include a systolic/diastolic blood pressure ≥ 130 and ≥ 80 mmHg, correspondingly, a self-reported hypertension diagnosis, or using antihypertensive medication. Smoking status was classified into current smokers (> 100 cigarettes in their lifetime and are currently smoking), former smokers (> 100 cigarettes in their lifetime but have since ceased smoking), and non-smokers (< 100 cigarettes in their lifetime). Similarly, alcohol consumption was classified into never drinkers (< 12 alcoholic drinks in their lifetime), low-to-moderate drinkers (one drink daily for women and two drinks daily for men throughout the previous year), and heavy drinkers (> one drink daily for women and > two drinks daily for men throughout the previous year). Energy intake was assessed by averaging two values obtained from two 24 h dietary recall interviews. BMI was categorized into underweight/normal (< 25 kg/m^2^), overweight (25–30 kg/m^2^), and obesity (≥ 30 kg/m^2^). Information regarding the use of Vit C supplements was obtained from the dietary supplements section and classified into three distinct categories: no supplementation, standalone Vit C supplements, and multivitamins containing Vit C. Physical activity data were collected using physical activity questionnaires, and metabolic equivalent (MET) scores were calculated based on the type and intensity of activities (20). According to the American Physical Activity Guidelines (21), we categorized physical activity into three levels: inactive (0 MET-min/week), insufficiently active (1–599 MET-min/week), and sufficiently active (≥ 600 MET-min/week). The DII was included as a covariate to account for the combined effects of pro-inflammatory, anti-inflammatory, and antioxidant dietary components, reflecting overall dietary inflammatory potential. It quantifies this potential by comparing intakes of 45 food parameters to a global reference database, with lower scores indicating a healthier diet pattern (22).

### Statistical analysis

Owing to the complex NHANES sampling design, sample weights were applied in the analyses to account for selection and non-response biases, ensuring that the findings accurately represent the general U.S. population. The Anderson–Darling test revealed that continuous variables deviated from a normal distribution. Consequently, they were reported as medians and interquartile ranges (IQR), while categorical variables were expressed as frequencies and weighted percentages. To assess differences in baseline features across groups, we employed the Kruskal–Wallis rank-sum test for continuous variables while using the Chi-Squared test for categorical variables.

Survey-weighted linear regression (LR) models were utilized to ascertain regression coefficients (*β*) and 95% confidence intervals (CI) for the connection between S-vit C levels and PhenoAgeAccel, utilizing three statistical models. Model 1 involved unadjusted analyses, whereas Model 2 accounted for age, sex, race, Marital status, and education level. The fully adjusted Model 3 was further adjusted for household income, smoking status, energy intake, alcohol consumption, BMI, diabetes, hypertension, Vit C supplements use, DII, and physical activity. Multicollinearity among covariates was assessed using variance inflation factors (VIF), with all VIFs < 5.0 indicating no significant collinearity. S-vit C levels were examined as both continuous and categorical variables, with participants stratified into quartiles according to their S-vit C concentrations. Linear trend analyses were conducted by incorporating the median value of each quartile of S-vit C levels as a continuous variable in the regression models. Additionally, we utilized restricted cubic spline (RCS) analysis to investigate the potential nonlinear relationship between continuous S-vit C levels and PhenoAgeAccel, with knot points for the RCS curve selected based on the optimization of the Akaike information criterion (AIC). Upon identifying a nonlinear relationship via RCS analysis, a two-piecewise LR model and a log-likelihood ratio test were utilized to evaluate the threshold effect and ascertain the inflection point through a recursive method (23).

Subgroup analyses were undertaken to evaluate stratification variables: age, sex, BMI, diabetes, and hypertension, particularly focusing on their interaction effects with Vit C levels. Two sensitivity analyses were conducted to evaluate the robustness of the primary findings. First, we utilized PhenoAgeAccel data obtained from the"BioAge"R package, which has developed and validated an alternative algorithm for assessing PhenoAge and PhenoAgeAccel (24). Additionally, we excluded data from the 2017–2018 cycle because it used a different testing method for C-reactive protein, which May introduce systematic bias. Participants with implausible DII or energy intake values, identified using the IQR method with thresholds set at 1.5 × IQR (below Q1−1.5 × IQR or above Q3 + 1.5 × IQR were flagged as implausible values), were also excluded to minimize bias due to misreporting (25).

Missing value imputation was not required, as we excluded participants having missing continuous data during the initial screening process, whereas those with missing categorical data were assigned to a “Missing” category. Statistical analyses were performed using a significance threshold of two-tailed *p* < 0.05. Data analyses were Carried out using Stata 17 (Stata Corporation, College Station, Texas, United States) and R 4.3.3 (R Core Team, Vienna, Austria).

## Results

### Baseline characteristics of participants

The baseline features of the 10,118 study participants, categorized into quartiles based on their S-vit C concentrations, indicated significant variations across demographic, socioeconomic, and health-related parameters (Table 1). Participants in the highest quartile tended to be older, were predominantly female, had better dietary habits, engaged in adequate physical activity, reported lower daily energy intake, achieved higher levels of education, and had higher household incomes. Additionally, this group exhibited a reduced incidence of smoking and heavy alcohol intake. Participants in the highest S-vit C quartile also demonstrated better health outcomes, characterized by reduced incidences of obesity, hypertension, and diabetes, compared to the lowest quartile.

**Table 1:** Baseline characteristics of participants

Characteristics	Quartiles of S-vit C concentrations (mg/dL)				*p*-value^2^
	Q1 (0.010–0.570) Weighted *N* = 41,362,103 Unweighted *n* = 2,495^1^	Q2 (0.570–0.915) Weighted *N* = 37,982,637 Unweighted *n* = 2562^1^	Q3 (0.915–1.190) Weighted *N* = 40,235,260 Unweighted *n* = 2,474^1^	Q4 (1.190–5.170) Weighted N = 44,086,413 Unweighted *n* = 2,587^1^	
Age (years)	43 (32, 54)	42 (31, 54)	44 (31, 55)	48 (34, 61)	< 0.001
Energy intake (kcal/day)	2022 (1533, 2710)	2114 (1536, 2786)	2088 (1597, 2719)	1969 (1516, 2542)	0.004
DII	2.31 (1.05, 3.27)	1.78 (0.26, 2.87)	1.38 (– 0.10, 2.67)	1.17 (– 0.35, 2.48)	< 0.001
PhenoAge (years)	43 (32, 55)	41 (29, 54)	41 (30, 53)	44 (30, 57)	< 0.001
PhenoAgeAccel (years)	– 0.6 (– 3.3, 3.4)	– 1.4 (– 4.5, 2.1)	– 2.5 (– 5.5, 0.6)	– 3.8 (– 6.6, – 0.7)	< 0.001
Sex, %					< 0.001
Male	1457 (57.8%)	1427 (56.0%)	1276 (52.2%)	993 (36.7%)	
Female	1038 (42.2%)	1135 (44.0%)	1198 (47.8%)	1594 (63.3%)	
Race, %					< 0.001
Mexican American	384 (6.8%)	578 (11.3%)	536 (10.1%)	454 (7.0%)	
Other Hispanic	113 (3.7%)	173 (6.1%)	156 (5.7%)	139 (4.6%)	
Non-Hispanic White	1204 (72.4%)	877 (60.2%)	970 (65.8%)	1273 (73.4%)	
Non-Hispanic Black	593 (10.4%)	672 (14.6%)	557 (10.9%)	463 (8.4%)	
Other Race	201 (6.7%)	262 (7.8%)	255 (7.6%)	258 (6.7%)	
Education level, %					< 0.001
< high school	695 (17.8%)	685 (16.1%)	546 (12.6%)	484 (10.4%)	
High school graduate	723 (33.8%)	605 (26.3%)	561 (23.8%)	590 (23.1%)	
> high school	1076 (48.4%)	1270 (57.5%)	1365 (63.6%)	1512 (66.5%)	
Missing	1 (< 0.1%)	2 (0.1%)	2 (< 0.1%)	1 (< 0.1%)	
Marital status, %					0.002
Married/living with partner	1475 (63.1%)	1584 (63.6%)	1583 (67.2%)	1660 (67.3%)	
Never married	444 (18.4%)	535 (20.9%)	473 (18.5%)	421 (15.5%)	
Widowed/divorced/separated	573 (18.4%)	443 (15.5%)	415 (14.2%)	505 (17.1%)	
Missing	3 (0.1%)	0 (0.0%)	3 (0.1%)	1 (< 0.1%)	
Household income, %					< 0.001
Low	781 (22.2%)	706 (19.9%)	564 (16.1%)	534 (13.2%)	
Moderate	917 (36.0%)	940 (34.8%)	903 (33.2%)	911 (32.9%)	
High	600 (36.0%)	742 (39.4%)	841 (45.4%)	980 (48.4%)	
Missing	197 (5.9%)	174 (5.9%)	166 (5.3%)	162 (5.5%)	
BMI category, %					< 0.001
Under/normal weight	611 (25.6%)	566 (23.9%)	725 (30.5%)	963 (40.8%)	
Overweight	708 (25.8%)	862 (31.5%)	889 (37.5%)	920 (34.1%)	
Obesity	1,176 (48.6%)	1,134 (44.7%)	860 (31.9%)	704 (25.1%)	
Alcohol consumption, %					< 0.001
Never drinkers	156 (4.9%)	188 (6.5%)	205 (6.6%)	221 (7.6%)	
Low-to-moderate drinkers	654 (29.4%)	768 (33.2%)	848 (36.8%)	938 (38.1%)	
Heavy drinkers	1,007 (43.0%)	943 (40.1%)	820 (37.1%)	761 (32.6%)	
Missing	678 (22.7%)	663 (20.1%)	601 (19.5%)	667 (21.7%)	
Smoking status, %					< 0.001
Current smokers	984 (39.1%)	561 (22.6%)	430 (17.6%)	346 (13.6%)	
Former smokers	524 (20.9%)	577 (24.2%)	599 (24.2%)	665 (25.4%)	
Non-smokers	987 (39.9%)	1424 (53.3%)	1445 (58.2%)	1575 (61.0%)	
Missing	0 (0.0%)	0 (0.0%)	0 (0.0%)	1 (< 0.1%)	
Vit C supplements use, %					< 0.001
Only Vit C	52 (2.2%)	112 (5.1%)	174 (7.3%)	448 (20.1%)	
Multivitamins containing Vit C	87 (3.8%)	197 (7.8%)	280 (12.3%)	338 (13.3%)	
No	2356 (94.0%)	2253 (87.1%)	2020 (80.4%)	1801 (66.6%)	
Physical activity, %					< 0.001
Inactive	261 (8.5%)	203 (5.9%)	160 (5.7%)	189 (6.0%)	
Insufficiently active	488 (20.5%)	495 (21.2%)	521 (21.4%)	545 (21.7%)	
Sufficiently active	1023 (44.7%)	1198 (50.4%)	1236 (55.2%)	1324 (55.8%)	
Missing	723 (26.4%)	666 (22.5%)	557 (17.7%)	529 (16.5%)	
Diabetes, %					< 0.001
Yes	464 (13.8%)	432 (12.1%)	293 (8.1%)	297 (8.1%)	
No	2031 (86.2%)	2130 (87.9%)	2181 (91.9%)	2290 (91.9%)	
Hypertension, %					< 0.001
Yes	1451 (52.7%)	1360 (50.4%)	1205 (43.6%)	1249 (43.8%)	
No	1044 (47.3%)	1202 (49.6%)	1269 (56.4%)	1338 (56.2%)	

^1^Continuous variables were presented as medians and interquartile ranges (Q1, Q3); categorical variables were presented as unweighted numbers and weighted percentages

^2^Kruskal-Wallis rank-sum test for continuous variables; Chi-Squared test with Rao & Scott's second-order correction for categorical variables

### Association between quartiles of S-vit C levels and PhenoAgeAccel

The results of the weighted LR models showed that in the unadjusted model, participants in the highest quartile of S-vit C concentrations had a 3.85-year reduction in PhenoAgeAccel (*β* =  − 3.85, 95% CI  − 4.36 to − 3.34, *p* < 0.001) compared to those in the lowest quartile. Upon adjustment for multiple covariates, the highest quartile of S-vit C concentrations continued to show a significant association with a lower PhenoAgeAccel in contrast to the lowest quartile (*β* =  − 3.64, 95% CI  − 4.12 to − 3.16, *p* < 0.001 in Model 2; *β* =  − 2.04, 95% CI  − 2.38 to − 1.69, *p* < 0.001 in Model 3). VIFs for all variables were less than 2.0, indicating no collinearity. In addition, tests for linear trends across quartiles of S-vit C levels yielded statistically significant results (Table 2).

**Table 2:** Association between quartiles of S-vit C concentrations and PhenoAgeAccel

S-vit C concentrations (mg/dL)	Models					
	1		2		3	
	*β* (95% CI)	*p*	*β* (95% CI)	*p*	*β* (95% CI)	*p*
Quartiles						
Q1 (0.010–0.570)	Reference		Reference		Reference	
Q2 (0.570–0.915)	– 1.11 (– 1.75, – 0.47)	0.001	– 1.16 (– 1.79, – 0.53)	< 0.001	– 0.68 (– 1.24, – 0.11)	0.020
Q3 (0.915–1.190)	– 2.54 (– 3.13, – 1.95)	< 0.001	– 2.44 (– 3.03, – 1.87)	< 0.001	– 1.30 (– 1.74, – 0.85)	< 0.001
Q4 (1.190–5.170)	– 3.85 (– 4.36, – 3.34)	< 0.001	– 3.64 (– 4.12, – 3.16)	< 0.001	– 2.04 (– 2.38, – 1.69)	< 0.001
*P* for trend		< 0.001		< 0.001		< 0.001

Model 1: Unadjusted

Model 2: Adjusted for age, sex, race, marital status, and education level

Model 3: Further adjusted for household income, smoking status, energy intake, alcohol consumption, BMI, diabetes, hypertension, Vit C supplements use, DII, and physical activity

### RCS analysis and threshold effect analysis

**Fig. 2:**
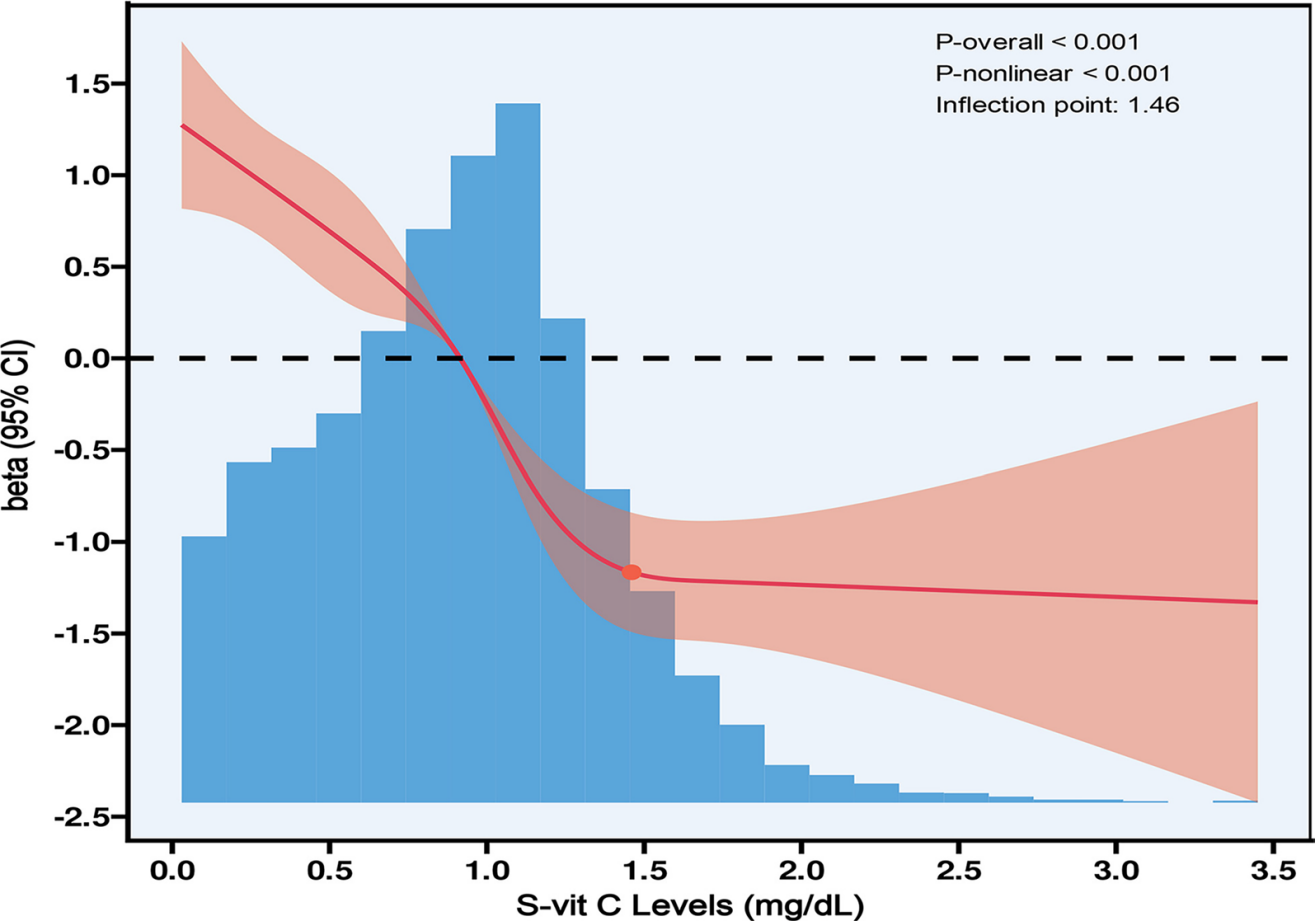
The RCS curve of the nonlinear connection between S-vit C concentrations and PhenoAgeAccel. Regression coefficients (*β*) adjusted for age, sex, race, marital status, education level, household income, smoking status, energy intake, alcohol consumption, BMI, diabetes, hypertension, Vit C supplements use, DII, and physical activity

When evaluating S-vit C concentrations as a continuous variable and adjusting for all covariates, the RCS curve Manifested a nonlinear relationship between S-vit C concentrations and PhenoAgeAccel. The knot points for the RCS curve between 3 and 7 were tested respectively, and the model with lowest AIC value was selected for RCS. Finally, we used RCS with 4 knots at the 5th, 35th, 65th and 95th percentiles (Fig. 2). The inflection point was identified at a S-vit C level of 1.46 mg/dL, with significant differences detected on either side of this threshold (log-likelihood ratio test, *p* < 0.001). For S-vit C levels below 1.46 mg/dL, an increase of 1 mg/dL was related to a decrease of 2.20-year in PhenoAgeAccel (*β* =  − 2.20, 95% CI  − 2.53 to − 1.87, *p* < 0.001). Conversely, for S-vit C levels above 1.46 mg/dL, no significant connection was observed (*β* = 0.47, 95% CI  − 0.36 to 1.31, *p* = 0.264) (Table 3).

**Table 3:** The outcome of the two-piecewise linear regression model

Two-piecewise linear regression	*β* (95% CI)	*p*
S-vit C concentrations (mg/dL)		
< 1.46 (*n* = 9041)	– 2.20 (– 2.53, – 1.87)	< 0.001
≥ 1.46 (*n* = 1077)	0.47 (– 0.36, 1.31)	0.264
* P* for the log-likelihood ratio test		< 0.001

Adjusted for age, sex, race, marital status, education level, household income, smoking status, energy intake, alcohol consumption, BMI, diabetes, hypertension, Vit C supplements use, DII, and physical activity

### Subgroup analyses and interaction effects tests

**Fig. 3:**
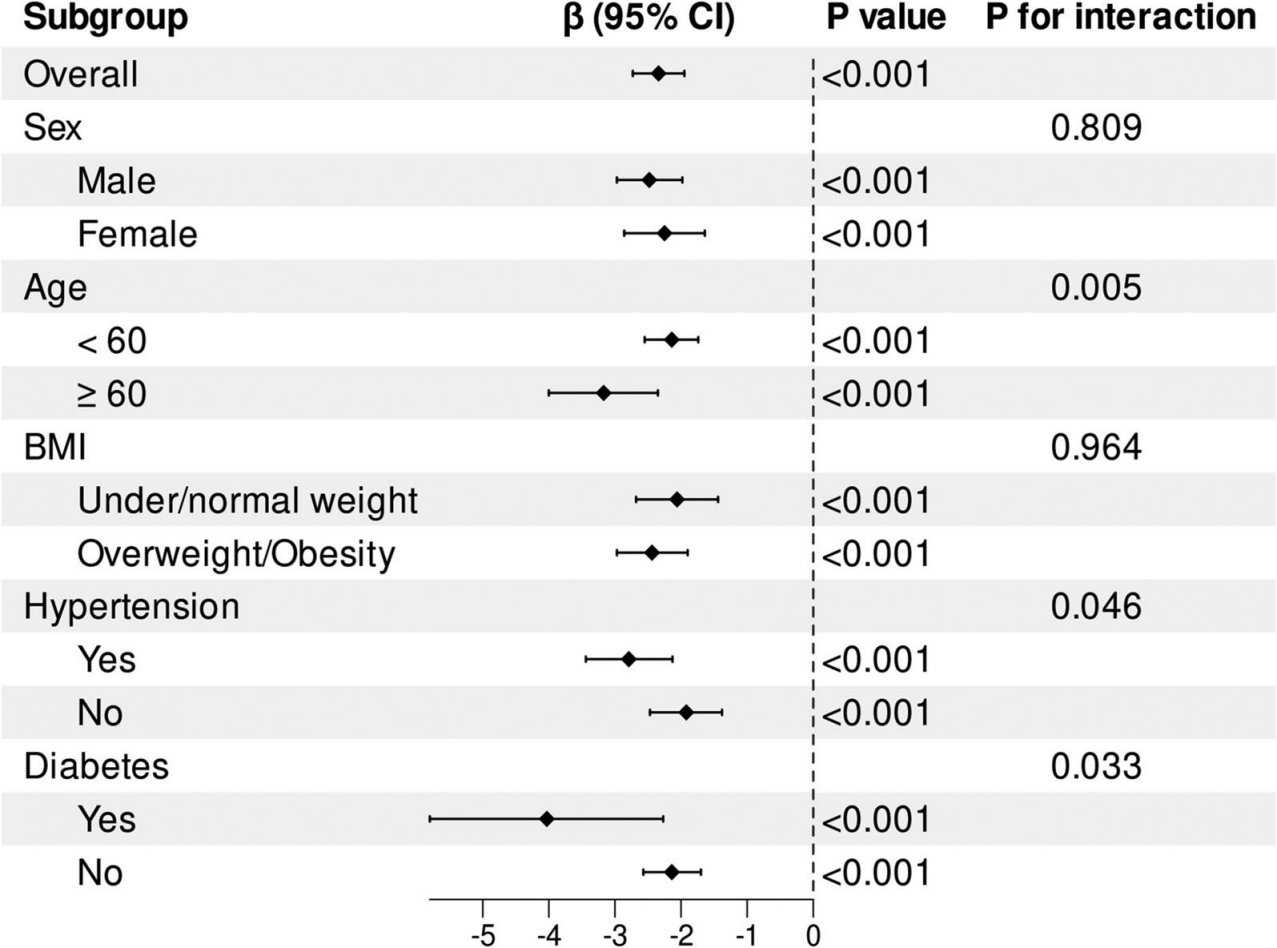
Subgroup analyses of the association between S-vit C concentrations and PhenoAgeAccel, stratified by BMI, sex, age, hypertension, and diabetes. Regression coefficients (*β*) adjusted for age, sex, race, marital status, education level, household income, smoking status, energy intake, alcohol consumption, BMI, diabetes, hypertension, Vit C supplements use, DII, and physical activity, excluding the stratifying factors

The subgroup analyses demonstrated that participants in the highest quartile of S-vit C concentrations consistently displayed lower PhenoAgeAccel compared to the lowest quartile across various demographic and health-related subgroups. Notably, there were significant interactions within the age, hypertension, and diabetes subgroups, indicating a stronger negative association between S-vit C levels and PhenoAgeAccel among participants aged ≥ 60 years, as well as those having hypertension or diabetes (Fig. 3).

### Sensitivity analyses

Sensitivity analyses conducted using PhenoAgeAccel data from the"BioAge"R package corroborated that S-vit C concentrations were inversely associated with PhenoAgeAccel. In the fully adjusted model, participants in the highest quartile of S-vit C concentrations showed a reduction of 1.42-year in PhenoAgeAccel (*β* =  − 1.42, 95% CI  − 1.74 to − 1.11, *p* < 0.001), compared to those in the lowest quartile (Table 4). When participants from the 2017–2018 cycle and those with implausible DII or energy intake values were excluded, the results remained robust (Table 5).

**Table 4:** Results of sensitivity analyses using PhenoAgeAccel data from the “BioAge” R package

	Models					
S-vit C concentrations (mg/dL)	1		2		3	
	*β* (95% CI)	*p*	*β* (95% CI)	*p*	*β* (95% CI)	*p*
Quartiles						
Q1 (0.010–0.570)	Reference		Reference		Reference	
Q2 (0.570–0.915)	– 0.99 (– 1.37, – 0.61)	< 0.001	– 0.86 (– 1.24, – 0.48)	< 0.001	– 0.49 (– 0.81, – 0.17)	0.003
Q3 (0.915–1.190)	– 2.24 (– 2.69, – 1.78)	< 0.001	– 1.96 (– 2.39, – 1.52)	< 0.001	– 0.99 (– 1.34, – 0.64)	< 0.001
Q4 (1.190–5.170)	– 3.39 (– 3.73, – 3.04)	< 0.001	– 2.78 (– 3.10, – 2.45)	< 0.001	– 1.42 (– 1.74, – 1.11)	< 0.001
*P* for trend		< 0.001		< 0.001		< 0.001

Model 1: Unadjusted

Model 2: Adjusted for age, sex, race, marital status, and education level

Model 3: Further adjusted for household income, smoking status, energy intake, alcohol consumption, BMI, diabetes, hypertension, Vit C supplements use, DII, and physical activity

**Table 5:** Results of sensitivity analyses by excluding participants from the 2017–2018 cycle and those with implausible values

	Models					
S-vit C concentrations (mg/dL)	1		2		3	
	*β* (95% CI)	*p*	*β* (95% CI)	*p*	*β* (95% CI)	*p*
Quartiles						
Q1 (0.010–0.570)	Reference		Reference		Reference	
Q2 (0.570–0.915)	– 0.87 (– 1.44, – 0.31)	0.004	– 0.84 (– 1.36, – 0.32)	0.002	– 0.44 (– 0.98, 0.11)	0.111
Q3 (0.915–1.190)	– 2.29 (– 2.72, – 1.85)	< 0.001	– 2.11 (– 2.54, – 1.69)	< 0.001	– 1.06 (– 1.51, – 0.62)	< 0.001
Q4 (1.190–5.170)	– 3.48 (– 3.97, – 2.99)	< 0.001	– 3.19 (– 3.64, – 2.74)	< 0.001	– 1.70 (– 2.10, – 1.30)	< 0.001
*P* for trend		< 0.001		< 0.001		< 0.001

Model 1: Unadjusted

Model 2: Adjusted for age, sex, race, marital status, and education level

Model 3: Further adjusted for household income, smoking status, energy intake, DII, alcohol consumption, BMI, diabetes, hypertension, Vit C supplements use, DII, and physical activity

## Discussion

The current study explored the connection between S-vit C concentrations and biological aging. The findings showed a strong inverse association between S-vit C concentrations and PhenoAgeAccel, a metric utilized to assess biological aging. Higher S-vit C concentrations were consistently related to reduced PhenoAgeAccel, suggesting that individuals having elevated S-vit C levels exhibit slower rates of biological aging. This inverse association was especially pronounced among older adults and individuals having chronic diseases, such as hypertension and diabetes. This bolsters the hypothesis that Vit C, a dietary antioxidant, may confer protective effects against aging and age-related diseases.

The anti-aging effects of Vit C may be mediated through several pathways. It neutralizes excess reactive oxygen species, thereby preventing oxidative damage to DNA, proteins, and lipids, which helps maintain cellular structure and function (26). Additionally, Vit C modulates various aging-related signaling pathways, including NF-κB and MAPK, contributing to the suppression of inflammation and the delay of cellular senescence (26). Moreover, it regenerates vitamin E, promotes glutathione synthesis, and strengthens the overall antioxidant defense system. Furthermore, Vit C may slow telomere shortening, thereby extending cellular lifespan and alleviating OxS and the aging process (17). Notably, Vit C directly modulates key PhenoAge biomarkers, such as suppressing C-reactive protein via NF-κB inhibition (16), preserving albumin synthesis by reducing hepatic OxS, and improving glucose metabolism through attenuation of insulin resistance (17), this may potentially influence PhenoAge calculation. However, the persistence of the association in sensitivity analyses using alternative PhenoAge algorithms supports Vit C's independent role in fundamental aging processes beyond mere biomarker modulation.

Our study identified a significant reduction in PhenoAgeAccel among participants in the highest quartile of S-vit C levels, with reductions of 3.85 years in the unadjusted model and 2.04 years in the fully adjusted model. This is consistent with prior research demonstrating the association between elevated antioxidant levels and healthier aging. For instance, He et al. have revealed a positive relationship between a higher composite dietary antioxidant index and delayed biological aging (13). Similarly, the inverse relationship observed in our study between S-vit C levels and biological aging aligns with findings from studies investigating other dietary antioxidants and their effects on aging (14). Notably, one study examining dietary nutrient intake and aging found no significant association between Vit C consumption and biological aging acceleration (27). This disparity may arise from the assessment method, as Vit C intake was measured through dietary recall interviews, possibly not precisely reflected serum levels of the vitamin.

The identified nonlinear relationship between S-vit C concentrations and PhenoAgeAccel is particularly significant. This relationship exhibits an inflection point at 1.46 mg/dL, which aligns with previous pharmacokinetic studies indicating that Vit C concentrations tend to plateau at approximately 1.0 to 1.5 mg/dL (28, 29). This plateau likely representing the physiological saturation threshold of Vit C's antioxidant capacity and its excretion dynamics. Antioxidant systems function in a dynamic equilibrium, beyond a certain threshold, excess Vit C may be rapidly excreted by the kidneys or recycled through redox interactions with glutathione, reducing its overall anti-aging efficacy (30). In certain contexts, high doses of Vit C may paradoxically exert pro-oxidant effects, potentially counteracting its benefits (31). It is therefore important to investigate the optimal concentration of Vit C and whether supplementation beyond a certain threshold will result in diminishing effects.

The 1.46 mg/dL threshold has direct clinical relevance. Our data show that approximately 90% of participants have S-vit C levels below this value, indicating a broad need for targeted interventions. To reach this level, dietary modifications—such as consuming at least five daily servings of Vit C-rich foods (e.g., citrus fruits, bell peppers, or broccoli)—are effective, providing 200–400 mg/day and achieving plasma concentrations of 1.0–1.5 mg/dL. For high-risk populations (e.g., older adults or individuals with chronic conditions and malabsorption), low-dose supplementation (200–500 mg/day) can safely increase S-vit C levels to 1.2–1.8 mg/dL (28, 29). However, surpassing this threshold is unlikely to confer further anti-aging benefits. Importantly, high-dose supplementation (> 1000 mg/day) Carries potential risks, including pro-oxidant effects in the presence of free iron and a 30–50% increase in urinary oxalate excretion, which may elevate the risk of oxalate nephropathy or kidney stones in susceptible individuals (e.g., those with a history of renal impairment) (31, 32). Clinicians should prioritize dietary strategies and reserve supplementation for correcting deficiencies, aiming for an S-vit C level of ≥ 1.46 mg/dL while avoiding doses exceeding 1000 mg/day unless clinically warranted.

Our subgroup analyses showcased that the link between S-vit C and PhenoAgeAccel was significantly more pronounced among participants aged 60 and older, as well as those with hypertension or diabetes. This finding is of particular significance, as older adults and individuals with chronic conditions are more vulnerable to OxS and inflammation (33), both of which can accelerate aging. Previous research has indicated that antioxidants, such as Vit C, may confer greater benefits to populations experiencing elevated levels of OxS (34, 35). It is plausible that, within these groups, Vit C's ability to neutralize free radicals and mitigate inflammation May decelerate the progression of biological aging, thereby offering potential avenues for preventive strategies in aging-related diseases. Clinically, these findings advocate for integrating Vit C status assessment into routine geriatric and chronic disease Management, with a target threshold of 1.46 mg/dL to optimize anti-aging benefits.

In addition, sensitivity analyses supported our findings and confirmed the inverse association between S-vit C and biological ageing. These additional analyses strengthen the validity and robustness of our findings and highlight the consistency of the association across different data sources and analytical methods.

The strength of our study lies in the large sample size, the adjustment for multiple confounding variables, and the comprehensive analysis of the relationship, including nonlinear association and threshold effect. Nevertheless, several limitations should be recognized. First, the cross-sectional design limits our ability to infer causality, and reverse causality remains a major concern. Specifically, accelerated aging may lead to lower S-vit C levels through age-related physiological decline (e.g., intestinal malabsorption, reduced cellular uptake) or increased utilization due to chronic OxS—rather than low Vit C being the initial driver of aging. Therefore, it is unclear whether low S-vit C contributes to biological aging or is a consequence of it. Future longitudinal studies measuring serial S-vit C and aging biomarkers, or randomized trials testing Vit C supplementation in high-risk populations (e.g., elderly), are needed to clarify the directionality of this relationship. Second, the exclusion of participants aged 80 years and older (due to top-coding of age in NHANES 2017–2018 cycle) limits our understanding of the association in the oldest age group, who May exhibit distinct aging trajectories and Vit C metabolism. Third, although we adjusted for Vit C supplements use in the analysis, the relatively low prevalence of dedicated Vit C supplementation restricted our ability to perform a sensitive comparative analysis between supplements users and non-users. Future studies with larger supplements-user cohorts are needed to elucidate potential differential effects of dietary versus supplemental Vit C sources. In addition, although various potential confounders were taken into account, residual confounders May still exist due to unmeasured or unknown variables that could affect the association between S-vit C and biological aging. Future research should address these confounding factors through more comprehensive study designs and more extensive data collection. Fourth, the methods for measuring C-reactive protein varied across NHANES cycles. However, sensitivity analyses excluding the 2017–2018 cycle and employing an alternative PhenoAge algorithm yielded consistent results. Despite this, concerns regarding the potential for systemic bias persist. Moreover, since PhenoAge is calculated based on multiple biomarkers, Vit C, due to its antioxidant and anti-inflammatory properties, may affect these biomarkers directly. This could partially mediate the observed association, rather than representing an entirely independent effect. Future studies using alternative aging metrics less dependent on clinical biomarkers, or employing mediation analysis, could help disentangle this relationship. Furthermore, as the findings were derived from a U.S. population, it is important to assess their generalizability to other regions with differing racial demographics, dietary patterns, genetic backgrounds, or health systems. Although the identified threshold (1.46 mg/dL) aligns with pharmacokinetic saturation points across populations, future well-designed longitudinal and prospective studies are needed to ensure accurate validation of these findings.

## Conclusion

In conclusion, elevated S-vit C concentrations are related to slower biological aging, particularly among older adults and individuals with chronic conditions. However, there is a clear threshold (1.46 mg/dL) beyond which elevated S-vit C concentrations no longer mitigate biological aging. These results further highlight the possible role of Vit C as a modulator of the aging process. Further studies should elucidate causality, reveal the underlying biological mechanisms, and establish optimal Vit C levels.

## Data Availability

No datasets were generated or analysed during the current study.

## Abbreviations

CA: Chronological age
BA: Biological age
PhenoAge: Phenotypic age
PhenoAgeAccel: Phenotypic age acceleration
OxS: Oxidative stress
Vit C: Vitamin C
S-vit C: Serum vitamin C
NHANES: National health and nutrition examination survey
BMI: Body mass index
MET: Metabolic equivalent
IQR: Interquartile range
LR: Linear regression
CI: Confidence interval
VIF: Variance inflation factor
RCS: Restricted cubic spline
AIC: Akaike information criterion
DII: Dietary inflammatory index
